# E-cadherin gene re-expression in chronic lymphocytic leukemia cells by HDAC inhibitors

**DOI:** 10.1186/1471-2407-13-88

**Published:** 2013-02-25

**Authors:** Gwen Jordaan, Wei Liao, Sanjai Sharma

**Affiliations:** 1 Division of Hematology-Oncology, Greater Los Angeles VA Healthcare Center, UCLA School of Medicine, 11301 Wilshire Blvd, 90073, Los Angeles, CA, USA

**Keywords:** CLL, E-cadherin, Aberrant splicing, Nonsense mediated decay, Chromatin modeling, HDAC inhibitors, Wnt pathway

## Abstract

**Background:**

The tumor suppressor gene E-cadherin gene is frequently silenced in chronic lymphocytic leukemia (CLL) cells and results in wnt-pathway activation. We analyzed the role of histone epigenetic modifications in E-cadherin gene silencing.

**Methods:**

CLL specimens were treated with histone deacetylase inhibitor (HDACi) MS-275 and analyzed for E-cadherin expression with western blot and RT-PCR analysis. The downstream effects of HDACi treated leukemic cells were studied by analyzing the effect on wnt-pathway signaling. HDACi induced alterations in E-cadherin splicing were investigated by transcript specific real time PCR analysis.

**Results:**

Treatment of CLL specimens with histone deacetylase inhibitors (HDACi) treatment resulted in an increase of the E-cadherin RNA transcript (5 to 119 fold increase, n=10) in eight out of ten CLL specimens indicating that this gene is down regulated by histone hypoacetylation in a majority of CLL specimens. The E-cadherin re-expression in CLL specimens was noted by western blot analysis as well. Besides epigenetic silencing another mechanism of E-cadherin inactivation is aberrant exon 11 splicing resulting in an alternatively spliced transcript that lacks exon 11 and is degraded by the non-sense mediated decay (NMD) pathway. Our chromatin immunoprecipitation experiments show that HDACi increased the acetylation of histones H3 and H4 in the E-cadherin promoter region. This also affected the E-cadherin exon 11 splicing pattern as HDACi treated CLL specimens preferentially expressed the correctly spliced transcript and not the exon 11 skipped aberrant transcript. The re-expressed E- cadherin binds to β-catenin with inhibition of the active wnt-beta-catenin pathway in these cells. This resulted in a down regulation of two wnt target genes, LEF and cyclinD1 and the wnt pathway reporter.

**Conclusion:**

The E-cadherin gene is epigenetically modified and hypoacetylated in CLL leukemic cells. Treatment of CLL specimens with HDACi MS-275 activates transcription from this silent gene with expression of more correctly spliced E-cadherin transcripts as compared to the aberrant exon11 skipped transcripts that in turn inhibits the wnt signaling pathway. The data highlights the role of epigenetic modifications in altering gene splicing patterns.

## Background

The wnt-β-catenin pathway is a pro-growth and survival pathway that is active in multiple tumor types including chronic lymphocytic leukemia (CLL) [1-3]. Activation of this pathway in CLL is the result of high wnt and frizzled expression [4] along with epigenetic down regulation of wnt pathway antagonist genes including secreted frizzled-related protein (SFRP) family members, WIF1, DKK3 and APC [5,6]. The binding of wnts to their cognate receptors results in inhibition of GSK3β phosphorylation of β-catenin and its degradation. Stabilized β-catenin then translocates to the nucleus and interacts with lymphoid-enhancing (LEF) and T cell (TCF) transcription factors to activate transcription of wnt-target genes that include myc, LEF, cyclinD1, Cox-2, matrix metalloproteinase family members etc. [7-11]. E-cadherin expression is also able to inhibit the β-catenin translocation to the nucleus as its intracytoplasmic domain binds β- catenin [12,13]. In a previous study we identified silencing of the E-cadherin gene in CLL specimens as an additional mechanism of wnt pathway activation [14] as the ectopic expression of E-cadherin was sufficient to inhibit the active wnt-β- catenin pathway. Frequent loss of function of E-cadherin in CLL specimens and the activation of the wnt pathway highlights its role in CLL biology [14]. Also as inhibition of the wnt pathway results in apoptosis of CLL cells this is an important pathway for developing treatment strategies for this disease [15,16].

Epigenetic modifications such as DNA methylation and histone modifications silence a number of genes and are involved in leukemia initiation and progression [17,18]. These modifications are heritable, reversible and alter expression patterns of a number of genes without altering any DNA sequences. In the case of chronic lymphocytic leukemia (CLL) a number of genes are reportedly silenced by epigenetic alterations including the wnt pathway inhibitor genes [5,6] and more recently micro RNA expression was found to be modulated by epigenetic changes [19]. The inhibitors of these DNA epigenetic modifications are promising anticancer agents that allow re- expression of silenced genes, cell cycle arrest and apoptosis [20,21]. Histone deacetylase inhibitors (HDACi) are an example of drugs that are able to reverse epigenetic events and have useful clinical activity in various hematopoietic malignancies [21-23] including CLL where exposure to these drugs results significant apoptosis [24-26]. They increase the acetylation of the major histones H3 and H4 by inhibiting the histone deacetylases (HDACs) which leads to change in overall compactness of the chromatin and promotes accessibility of the DNA to the transcription factors and gene transcription [22].

In this report we explored the possibility that E-cadherin down regulation could be due to epigenetic modifications and studied the effect of HDACi on CLL specimens. Our previous report indicates that E-cadherin down regulation in CLL could be due to excessive aberrant splicing resulting in an alternatively spliced, non-functional E- cadherin transcript that lacks exon 11 of the gene [14]. This non-functional transcript has a premature termination codon and is degraded by the NMD pathway [27,28]. We explored this issue of aberrant splicing in the HDACi treated CLL specimens as well.

## Methods

### Cell culture and reagents

CLL specimens were obtained from CLL patients at the West Los Angeles VA hospital clinic after informed consent and an approval by the West Los Angles VA Medical Center Institutional Review Board. CLL cells were isolated by Ficoll-Paque gradient (GE healthcare, Piscataway, NJ) as manufacturer’s protocol and stored in liquid nitrogen. Specimens selected for analysis had more than 90% CLL cells in the PBMC isolate. For control samples, peripheral blood was obtained from four normal donors and PBMC (peripheral blood mononuclear cells) cells were isolated. HDACi MS-275 was obtained from ChemieTek, Indianapolis, IN and dissolved in DMSO. For HDACi treatment stored CLL specimens were taken in culture in RPMI1640 media with 10% fetal calf serum (complete media) and HDACi was added for 48 hours. To inhibit the NMD pathway and treat CLL cells with HDACi, cells were first treated with HDACi for 48 hours and emetine was added at a final concentration of 10μg/ml for the last eight hours. As a control, cells were treated with emetine alone for 8 hours. Annexin assay for analyzing apoptotic cells after HDACi treatment was performed with the Annexin V Apoptosis Detection kit II (BD Pharmingen, CA) as per manufacturer protocol.

### Western blot and immunoprecipitation analysis

For western blot analysis cells were washed with cold PBS and disrupted in lysis buffer (Cell Signaling, MA) supplemented with protease inhibitor cocktail (Thermo Fisher Scientific, MA). They were lysed on ice and then sonicated for 10 seconds. Insoluble material was removed by centrifugation (10,000 g, 10 min) and protein concentrations determined by BioRad DC protein assay (Hercules, CA). Samples were mixed with SDS sample buffer and 20–30 μg aliquots resolved on SDS/PAGE gels. Following transfer to PVDF membranes immunodetection was performed with E-cadherin (BD Pharmingen, CA), Ac H3 (06–599, Millipore), Ac-H4 antibody (sc-34263, Santa Cruz Biotechnology).

Detection was performed with horseradish peroxidase-conjugated secondary antibodies and chemiluminescence (ECL plus, GE Healthcare and LAS Mini imager, Fuji). Immunoprecipitation with beta-catenin antibody (Cell Signaling, MA) was performed with the Classic IP kit by (Thermo Fisher Scientific, MA).

### Real time PCR and transcript specific PCR analysis

E-cadherin expression in cells was analyzed by real time PCR analysis. The wild type E-cadherin transcript was quantified by real time PCR with the 5’ primer GGATGTGCTGGATGTGAATG that localizes to exon 10 of the E-cadherin gene, the 3’ primer CACATCAGACAGGATCAGCAGAA localizes to the exon 12 and the taqman probe TAACATATCGGATTTGGAGAGAC for the wild type E- cadherin transcript level, binds to the junction of exon 10-exon 11. The expression level of the skipped or aberrant transcript (transcript lacking exon 11) was determined by a 5’ primer that is at the junction of exon 10 and 12 (TATGGAACAGAAAATAACGTTC) and a 3’ primer in the exon 12 (TGTCATTCACATCAGACAGGAT) with a taqman probe (AACAGGGACACTTCTG). This primer set only amplifies the exon 11 skipped or the aberrant transcript. The ratio of these two transcripts in cells was calculated by the difference in their Ct values. Taqman probes for HDAC 1–9, LEF1 and cyclin D1 (Applied Biosystems, CA) were used to determine their relative expression in CLL and PBMC cells. Real time PCR for actin expression was used as a control and relative expression was determined by the method of Pfall [29].

### Chromatin immunoprecipitation assay

ChIP assays were performed according to protocols of the EZ-ChIPTM Chromatin Immunoprecipitation kit (Millipore, MA). Briefly, 1×106 CLL cells were fixed with 1% formaldehyde for 10 min at 37°C. The cells were washed extensively with PBS, and the chromatin was sheared by sonication (Branson sonicator) to 200–400 bp fragments. The cross-linked histone-DNA complex was immunoprecipitated with anti pan Acetylated-H4 (sc-34263, Santa Cruz Biotechnology), anti pan Acetylated-H3 antibodies (06–599, Millipore). Normal mouse IgG was used as negative controls. DNA was obtained from the cross-linked complex and was amplified by a SYBR green based real-time PCR (Applied Biosystems, CA reagents) with specific primer sets. Primer sequences will be made available on request. Data sets were normalized to ChIP input values. The percent of input DNA immunoprecipitated by each antibody was calculated using the following method: % input = 100*2^Ctinput – CtIP^. (Ct value of input DNA-Ct value of immunoprecipitated DNA).

### Wnt reporter assay

Wnt activity in CLL specimens was determined by a luciferase reporter assay kit (SABiosciences, CA). This kit consists of a reporter construct expressing firefly luciferase with TCF/LEF binding sites and an identical negative control vector lacking TCF/LEF binding sites. Both constructs were transfected with a constitutively active renilla luciferase construct to control for transfection efficiency. CLL cells were thawed, cultured in complete medium for 24 hours, and then transfected by the Amaxa protocol (Nucleofector kit V, program U07). 8 hours after transfection cells transfected with the reporter construct were split into three wells. One well was left untreated and the other two were treated with either HDACi MS-275 (1μM concentration) or the GSK-3β inhibitor SB-216763 (1μM concentration) for another 24 hours. Cells were then lysed and analyzed by a Dual luciferase assay (Promega, Madison, WI). Data expressed as ratios of firefly to renilla to control for transfection efficiency.

## Results

### E-cadherin expression with HDAC inhibitors

**Figure 1:**
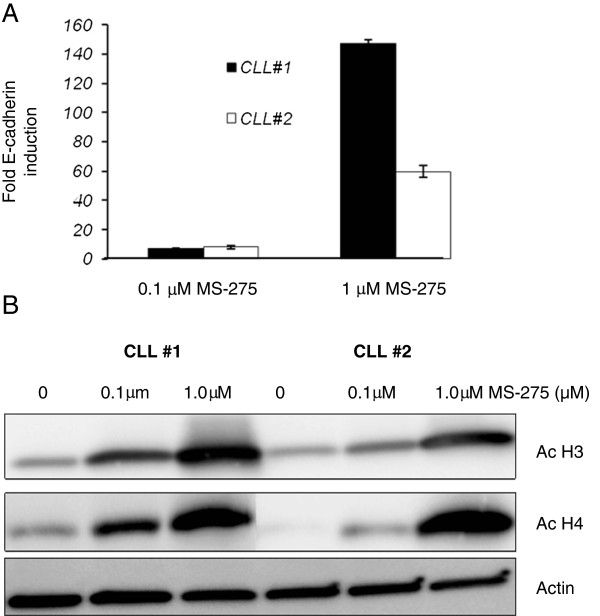
**Effect of HDAC inhibitors on the E-cadherin transcripts. A**. RT-PCR analysis to determine the change in expression of wild type E-cadherin transcripts with HDACi MS-275 treatment. Two primary CLL specimens were treated with two different concentrations of HDACi MS-275 (0.1μM and 1.0μM for 48 hours). (Data from two experiments, mean and SD). **B**. Western blot analysis of two CLL specimens that were treated with HDACi MS-275 (1μM for 48 hours). Lysates analyzed for acetylated histones H3 (Ac-H3), acetylated histone H4 antibodies (Ac-H4) with antibodies that recognize the acetylated histones and actin control.

Inhibition of the histone deacetylase activity is expected to result in an overall increase in histone acetylation and gene transcription [21]. To test whether E-cadherin expression in CLL is affected by histone acetylation status, CLL specimens were treated with HDAC inhibitors (HDACi). Initially two CLL specimens were treated MS-275 (Entinostat), a Class 1 HDAC inhibitor at two different concentrations for 48 hours and E-cadherin RNA expression was determined by real time PCR analysis. Control cells were treated with DMSO alone. Figure 1A shows the relative fold-increase in E-cadherin RNA expression (adjusted to actin, mean ± SD) by real time RT-PCR (untreated sample given an arbitrary value of 1). The HDACi increased E-cadherin RNA expression in the two CLL specimens as compared to the DMSO treated cells. For these two CLL specimens the fold increase in the E- cadherin RNA expression at 1.0 μM MS-275 concentration was between 60 and 150 fold with a smaller induction observed at the 0.1μM concentration.

To determine changes in the global acetylation status of CLL cells with HDACi treatment, a western blot analysis was performed. Two CLL specimens were treated with HDACi MS-275 at two different concentrations of 0.1 and 1.0 μM for 48 hours. Lysates were analyzed with acetylation specific pan acetyl-H3 and a pan acetyl-H4 antibodies. H3 and H4 are the two major histones that are post-translationally modified in the cells. As shown in Figure 1B, there is an increase in the acetylated histones in the two CLL specimens tested with HDACi MS-275.

### Effect of HDACi on E-cadherin exon 11 splicing

**Figure 2:**
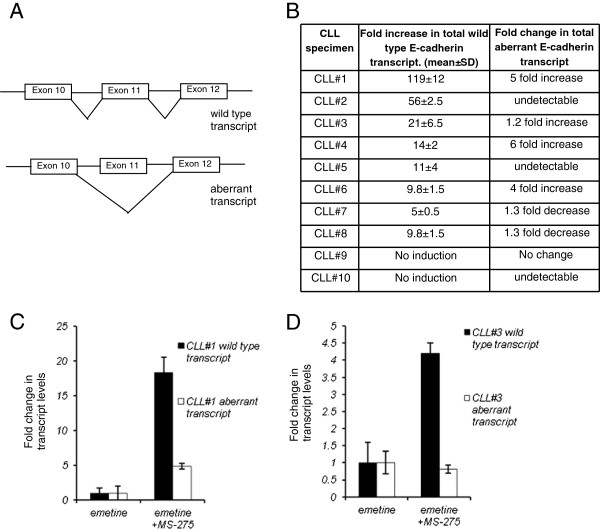
**Effect of HDACi on E-cadherin exon 11 splicing. A**. Schematic showing the wild type and exon 11 skipped aberrant transcript. Transcript specific PCR strategy is described in the Material and Methods section. **B**. Table showing the fold change in E-cadherin wild type RNA expression and exon 11 skipped RNA expression with HDACi treatment. (Data from at least two experiments for ten primary CLL specimens) **C**. **D**. Effect of NMD inhibition on HDACi mediated induction of transcripts. Two CLL specimens (CLL#1 and #3) were treated with either emetine alone for 8 hours or with MS-275 (1μM for 48 hours) and in the last 8 hours of MS-275 treatment emetine was added for 8 hours. Bar diagram shows the change in total transcript levels with MS-275 + emetine treatment as compared to the transcript levels in CLL specimens that were treated with emetine only.

With our finding of an increase in the wild type E-cadherin transcript (Figure 1A) with exposure to HDACi, we next investigated whether change in splicing contributes to this increase in E-cadherin expression [14]. There is a strong rationale for the HDACi role in splicing as they alter chromatin structure, increase promoter transcriptional activity and have a role in the selection of alternative exons [30-33]. A transcript specific real time PCR analysis was performed on HDACi treated CLL specimens (1.0μM, 48 hours) to quantify the correctly spliced or wild type transcript and the exon 11 skipped or aberrant transcripts (Figure 2A schematic and Material and Methods) and the data both for E-cadherin induction with HDAC MS-275 and the change in aberrant transcript is shown in Figure 2B Table. The data in this table represents total transcripts adjusted to actin and shows that E-cadherin transcript increases 5 to 119 fold as compared to the non-HDACi treated CLL cells. A significant specimen to specimen variation is observed as expected as these are primary leukemic cells from different CLL patients. In two of the ten CLL specimens (#9 and #10), no E- cadherin induction was observed with HDACi exposure. The HDACi effect on the aberrant transcript (Figure 2B Table) shows that in some CLL specimens even though we observed an increase in wild type E-cadherin expression, the aberrant transcript was not detectable by the assay and is suppressed (CLL#2 and #5). In two CLL specimens #7and #8 a decrease in the total aberrant transcript was observed while there is an induction in wild type transcript. Three CLL specimens increase the total aberrant transcript levels with HDACi treatment but in all the cases, this fold induction is less than the fold induction of the wild type transcript (CLL#1, #4, #6). Overall the HDACi exposure increases E-cadherin transcript and changes the ratio of the two transcripts in a majority of CLL specimens.

The NMD pathway is constitutively active in all cells including CLL specimens degrades the exon 11 skipped aberrant transcripts and the data shown above was obtained with these aberrant RNAs undergoing degradation by this pathway. Therefore to exclude the possibility that the changes in aberrant transcript are simply due to HDACi altering this degradation pathway, experiments were performed with a combination of HDACi and NMD blockade with a translational inhibitor, emetine to quantify the exon 11 skipped transcripts. Cells were treated with HDACi MS-275 (1.0 μM concentration) for forty eight-hours and emetine was added for the last eight hours of HDACi incubation to block the NMD followed by transcript specific PCR analysis. As a control, CLL specimen was treated with emetine alone for eight hours. Figure 2C,D shows a bar diagram from CLL #1 and #3, the total transcript levels with eight hour of emetine only treatment are assigned an arbitrary expression value of 1. In both the CLL specimens with MS-275 and emetine exposure we observe a difference in the induction of the two transcripts as compared to the emetine only treated cells. In CLL#1 (Figure 2C) the wild type transcript increases by 18-fold with a 5-fold increase in the aberrant transcript while in CLL specimen #3 (Figure 2D) interestingly there is a decrease in the total aberrant transcript with HDACi treatment with emetine while the wild type transcript increases four-fold. As the NMD blocker emetine is toxic to cells the overall induction observed with HDACi is lower as compared to induction in cells that were treated with HDACi alone (Figure 2B Table, CLL#1 and #3). The results demonstrate that the transcriptional induction with HDACi favors a selective expression of the correctly spliced E-cadherin transcript in the presence or absence of the NMD pathway.

### E-cadherin expression analysis by western blot analysis

**Figure 3:**
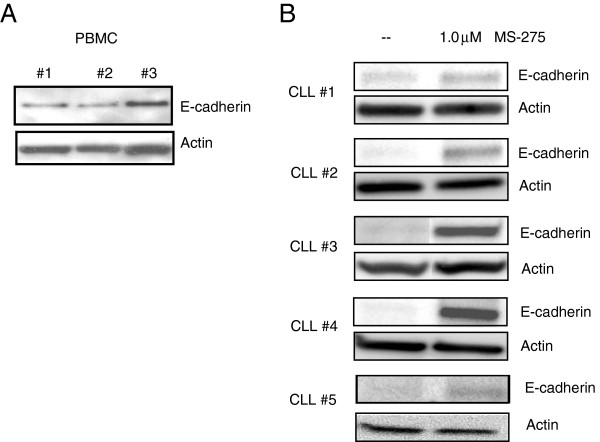
**Western Blot analysis for E-cadherin expression in PBMC and CLL specimens. A**. Three normal PBMC specimens were analyzed for E-cadherin expression. **B**. Western blot analysis of five representative CLL specimens of a total of eight CLL specimens that showed an increase in E-cadherin RNA. CLL specimens were treated with 1.0μM MS-275 for 48 hours and western blot was performed.

Loss of E-cadherin RNA expression in CLL specimens has been reported earlier [14]. To compare the E- cadherin expression between normal PBMC cells and CLL cells at the protein level a western blot analysis was performed. Figure 3A shows a western blot analysis of three different PBMC specimens from normal donors. The E-cadherin expression in PBMC is higher than the low to absent expression observed in the representative five CLL specimens shown in Figure 3B (non-treated control). To confirm that there is also an increase in E-cadherin protein expression, lysates from HDACi treated (MS-275 treated CLL specimens 1.0 μM, 48 hours) cells were analyzed for E-cadherin expression. There is a variable E-cadherin induction observed in all the CLL specimens tested by western blot analysis (Figure 3B) however in some cases the increase in expression on the western blot does not correlate with the fold increase in E-cadherin RNA expression. The HDACi MS-275 is thus able to re-express this tumor suppressor gene on both the RNA and protein level in a majority of CLL specimens and confirms that the silencing of this gene in CLL is in part due to epigenetic histone hypoacetylation.

### ChIP analysis of the E-cadherin promoter and HDAC expression

**Figure 4:**
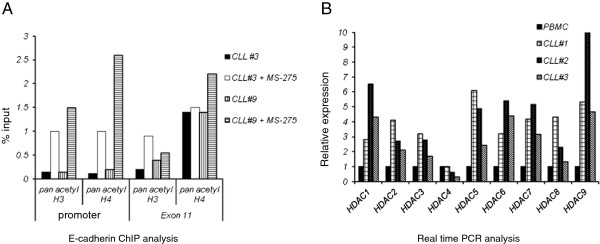
**Chromatin Immunoprecipitation analysis (ChIP) and HDAC expression analysis. A**. ChIP analysis of two CLL specimens (± HDACi). Cells were treated with MS-275 at a concentration of 1μM for 24 hours and sheared chromatin immunoprecipitated with two different antibodies, pan acetyl-H3 and pan acetyl-H4 followed by PCR amplification of the E-cadherin promoter and exon 11 regions. Real time PCR was performed to determine the percentage of input DNA (mean ± SD, data from two experiments) immunoprecipitated by the acetylated histone antibodies **B**. Real time PCR analysis data on the relative expression level of HDAC1-9 in three CLL specimens relative to control PBMC RNA, data also adjusted to actin.

The western blot analysis (Figure 1) shows a global increase in the acetylated histones when CLL specimens are treated with HDACi. To further confirm the acetylation change at the E- cadherin promoter a ChIP analysis was performed. Two CLL specimens were analyzed with two different antibodies that only immunoprecipitate the acetylated histone H3 and H4. E-cadherin promoter and the exon 11 of the E-cadherin gene were amplified by PCR with specific primers. The exon 11 region of E-cadherin was also analyzed by the ChIP assay to determine whether there are any specific epigenetic alterations that contribute to improper splicing of this exon. A sensitive real time PCR strategy was employed to determine the changes in the immunoprecipitated DNA relative to the input DNA. The results in Figure 4A show that both acetylated histone H3 and H4 are increased at the E-cadherin promoter region in cells that were treated with HDACi MS-275 (mean ± SD from two independent experiments). The results for the exon 11 region ChIP assay show a smaller increase in acetylated H3 and H4 histones as compared to the E-cadherin promoter region indicating that histone acetylation with HDACi are more pronounced at the E-cadherin promoter region as compared to the exon 11 region.

Histone hypoacetylation and silencing of the genes is due to an over expression of HDACs, enzymes that deacetylate the histones. A number of HDAC genes are expressed in cells and their expression was analyzed in three CLL specimens. Figure 4B shows the HDAC 1–9 expression in CLL specimens as compared to normal PBMC with data adjusted to actin. The data shows that except for HDAC4, all the other HDAC family of genes tested are significantly overexpressed in CLL specimens.

### Effect of E-cadherin expression on apoptosis and the wnt pathway

**Figure 5:**
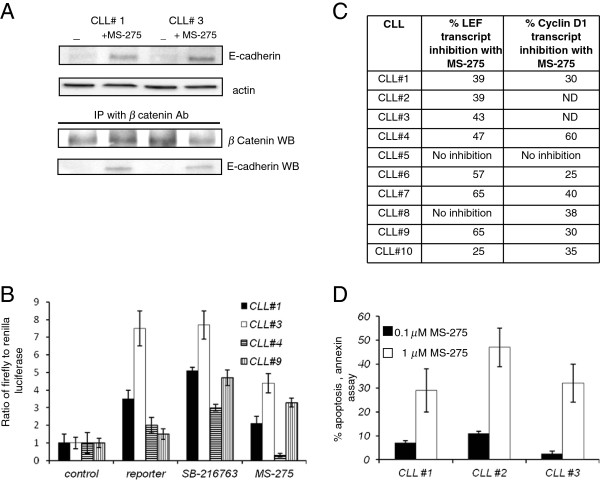
**Functional effects of HDACi on CLL specimens A.** Two CLL specimens were treated with HDACi and western blot and immunoprecipitation analysis was performed. Top panel is an E-cadherin western blot with actin control showing the E- cadherin induction with HDACi exposure (MS-275 1.0μM, 48 hours). Bottom panel is the western blot analysis (WB) of the β-catenin immunoprecipitation material probed with anti-β-catenin antibody and anti-E-cadherin antibody. E-cadherin protein signal is observed in the beta-catenin immunoprecipitate material from HDACi treated CLL specimen. **B**. Wnt pathway luciferase reporter assay in CLL specimens. CLL specimens were transfected with the control or reporter constructs. Reporter transfected cells were separated in three groups, no treatment, SB-216763 compound or HDACi treatment. Data shown is a ratio of firefly to renilla. **C**. Table with real time PCR data with expression of LEF and cyclinD1 in CLL specimens treated with HDACi, MS-275. The numbers represent the percentage decrease in the expression of LEF and cyclinD1 in HDACi treated cells as compared to the non-treated control. **D**. Induction of apoptosis in CLL specimens with MS-275 treatment. Three CLL specimens were treated with two different concentration of HDACi MS-275 (0.1 and 1.0 μM concentration) and analyzed for induction of apoptosis at the 48 hour time point by a flow cytometry based annexin assay. (mean of three experiments).

A number of studies have reported that HDACi have growth inhibitory activity in CLL specimens [23-26] however the precise mechanism of action of these drugs is not known. As inhibition of wnt pathway results in apoptosis in CLL cells and E-cadherin expression inhibits this pathway [14], we next investigated the effect of HDACi on the wnt-β-catenin pathway in CLL specimens. E-cadherin mediated inhibition of the wnt pathway is reportedly due to its ability to sequestrate β-catenin so to determine whether this was also the case in CLL, two CLL specimens were treated with HDACi MS-275 (1μM, twenty-four hours). E-cadherin induction was confirmed as shown earlier (Figure 3B) and in Figure 5A, upper panels. This lysate was then subjected to immunoprecipitation with an anti-β-catenin antibody followed by western blot analysis (Figure 5A lower panel). β-catenin signal was detected in the immunoprecipitated material in the treated and the un-treated CLL specimens. We also observed the E- cadherin protein signal was also detected in the HDACi treated immunoprecipitate. The results indicate an association between the re-expressed E- cadherin and β-catenin in CLL specimens that are treated with HDACi MS-275. No E- cadherin or β-catenin signal was observed in the bead only control immunoprecipitation.

A downstream effect of this sequestration of β-catenin by E-cadherin is wnt-β- catenin pathway inhibition [1] and this was further analyzed by a wnt pathway reporter assay in CLL specimens that were treated with MS-275. CLL specimens were transiently transfected with the Amaxa nucleofection protocol and in four CLL specimens tested, an increase in wnt reporter activity (range of 1.5-7 fold) as compared the control reporter plasmid was observed (Figure 5B) [4]. As a positive control, transfected cells were also treated with the compound SB-216763 (1μM concentration), a GSK3β inhibitor that inhibits GSK3β and increases cellular wnt activity [4]. All the CLL specimens tested increased the wnt reporter activity in response to GSK3β inhibitor providing independent validation to the wnt reporter assay. The wnt reporter activity decreased in three of the four CLL specimens that were treated with MS-275 (1 M for 24 hours) demonstrating wnt pathway inhibition.

Inhibition of wnt pathway activity due to E-cadherin re-expression and HDACi exposure is also expected to alter the expression of wnt pathway target genes. To further confirm the wnt pathway inhibition, we analyzed two well-described wnt target genes, LEF (lymphoid enhancing factor) and cyclinD1 [4] that are also upregulated in CLL. We first compared the expression of LEF and cyclin D1 in normal peripheral blood B cells and CLL specimens by real time PCR analysis. Both these genes were upregulated in all the CLL specimens tested as compared to peripheral blood mononuclear B cells (data not shown). CLL specimens were then treated with HDACi, MS-275 (1 μM for 48 hours) and analyzed by real time RT-PCR analysis. LEF expression was downregulated in eight of ten CLL specimens tested (Figure 5C table). In the case of cyclin D1, seven out of eight CLL specimens down regulated cyclin D1 expression (Figure 5C table) when exposed to HDACi. Downregulation of wnt reporter activity and two well known wnt target genes with HDACi support the role of HDACi in the inhibition of the wnt-beta-catenin pathway.

To further analyze the effect of HDACi on CLL specimens, an apoptosis assay was performed. Three CLL specimens were analyzed with a flow cytometry based annexin V assay. Cells were treated with two different concentrations of HDACi for 48 hours and analyzed for annexin staining as described. At the 1 m concentration of MS-275, 30-45% CLL cells were found to be annexin positive or apoptotic. Similar findings have been reported with other HDACi as well [23-26]. Overall the findings indicate an inhibition of the wnt pathway in CLL specimens with HDACi mediated E-cadherin re- expression.

## Discussion

Our study with primary CLL specimens demonstrates that the frequent loss of E- cadherin gene expression in CLL specimens is due to histone epigenetic silencing of the E-cadherin promoter. This gene silencing can be reversed by HDACi that acetylate the histones in the promoter region of this gene and activate E-cadherin gene transcription. Furthermore this HDACi induced E-cadherin expression inhibits the wnt- β-catenin pathway in CLL by interacting with β-catenin and thereby inhibiting its transactivation function. An additional interesting finding of the study is the effect of HDACi on E-cadherin splicing as there is a preferential expression of correctly spliced E-cadherin transcripts.

HDACi increase the E-cadherin expression in a majority of CLL specimens as determined by real time PCR and this increase in the transcript is also translated into an increase in E-cadherin signal on the western blot analysis. The induction of E-cadherin expression by western blot analysis is variable and a potential reason could be the pro-apoptotic effect of the HDACi that inhibits protein translation in some CLL specimens. Lack of response to HDACi in two out of ten CLL specimens could be due to other mechanisms including E-cadherin promoter methylation that has been reported [34]. With this significant increase in wild type E-cadherin RNA transcripts with HDACi we investigated whether there was a qualitative change in the RNA transcripts as a number of cellular alterations induced by HDACi such as gene transcription, change in chromatin structure are also known to alter gene splicing patterns [30-32].

To study this we analyzed the HDACi treated CLL specimens for the two E- cadherin transcripts. Analysis of a number of CLL specimens shows a variable response of the exon 11 skipped transcripts to HDACi exposure. In some CLL specimens there is a complete loss of expression of this transcript and in some cases a decrease in expression of aberrant transcript while the total wild type transcript consistently increased in amount in the majority of CLL specimens (Figure 2C). In two CLL specimens the absolute amount of aberrant transcript also increases with HDACi exposure albeit the fold induction is less than the wild type transcript can be explained by a more robust HDACi effect on overall E-cadherin transcription than its ability to alter aberrant exon 11 splicing. Overall we observe a much smaller increase in the exon 11 skipped transcripts as compared to the correctly spliced transcript with HDACi exposure. This change in the ratio of the two transcripts is not due to a change in the activity of the NMD pathway as experiments with NMD blocker emetine and HDACi show similar change in ratios. With NMD blockade as there is no degradation of the aberrant transcript, both the transcripts should have a parallel increase in fold induction. The lack of similar fold induction of the two transcripts in the two CLL specimens with NMD blockade supports the argument that with HDACi there is a qualitative change in splicing pattern along with transcriptional induction.

Histones play an important role in splicing, they are positioned in a non-random manner on the genome with a higher concentration at the exon-intron junction and certain histone modifications are preferentially seen in the exons as compared to the surrounding introns [35]. Histone epigenetic alterations are mainly found in the promoter regions but can also be detected in exonic regions [36,37]. With more than 95% of genes undergoing alternative splicing [38,39] in the human genome epigenetic histone deacetylation and other modifications such as methylation in cancer cells can alter splicing for the vast majority of genes and thereby alter the sequences of many RNA transcripts. This alteration in splicing can result in RNA isoforms and proteins with entirely different biological characteristics and is frequently observed in cancer cells [40,41]. For example the Bcl-x transcript is alternatively spliced to produce the anti-apoptotic Bcl-x (L) or the proapoptotic Bcl-x(s) and cancer cells often up regulate the anti-apoptotic Bcl-x (L) isoform which is associated with reduced sensitivity to chemotherapeutic drugs [42]. The malignant cells therefore have the ability to modulate alternative splicing for their growth and survival advantage.

This change in splicing pattern could be due to many HDACi effects on the chromatin and gene transcription [30,31]. There is a change in chromatin compactness that in turn alters accessibility of DNA to transcription and splicing factors [22]. It is also plausible that certain splicing factors are involved in this splicing defect and their expression is altered by HDACi exposure [43]. Also as splicing and transcription occur simultaneously, changes in the transcription with HDACi would affect splicing as well. As HDACi clearly increase gene transcription of epigenetically silenced genes, this alone could change the splicing pattern of the E-cadherin gene. Studies have also shown that rate of transcriptional elongation can regulate splicing as well [reviewed in 30]. RNA Pol II promoter has a large C-terminal domain (CTD) that plays a role in splicing as its truncation results in splicing defects [30]. The CTD is also subject to regulation by phosphorylation and functions as a docking protein for splicing factors as well [30]. Differences in Pol II promoter structure thus results in changes in splicing [44-46]. It is possible that the change in ratio of the two transcripts observed with HDACi in CLL specimens is predominantly due to change in E-cadherin transcription. As the change in transcription function of the HDACi cannot be separated from its effect on chromatin compactness and splicing factors, the precise mechanism by which HDACI induce splicing alteration in CLL is not clear.

Mutations of the spliceosome component SF3B1 in CLL cells have been recently identified in 7.5-15% of CLL specimens [47]. It is proposed that these mutations could result in defective spliceosome function, dysregulated splicing, alterations in gene expression and isoform switch. The precise function of this mutation in CLL is still unclear but it is known that the mutations are associated with CLL specimens that are resistant to fludarabine [48]. Further investigation of gene splicing in CLL cells is thus increasingly important to understand how the various RNA isoforms affect the biology of CLL and to develop treatment approaches to modify the splicing.

The role and the mechanism E-cadherin silencing in hematopoietic cells is not well known. Our findings clearly indicate that one of the mechanisms of the loss of its expression is histone hypoacetylation. The HDACi increase the acetylation of histones in the region of the E-cadherin promoter as determined by the ChIP assay and appears that this alteration is more pronounced as compared to the acetylation change at the exon 11 region. The overexpression of HDACs in CLL specimens also supports role of this epigenetic alteration in CLL. A recent report has analyzed HDAC expression in CLL specimens and correlated with prognosis [49]. This report also describes upregulation of a number of different HDAC genes in CLL.

The re-expression of E-cadherin tumor suppressor gene can have a multitude of effects on tumor cells including adhesion, invasion, modulation of receptor kinase activity and wnt pathway activation [50]. These downstream effects are better defined in the epithelial cells as compared to the hematopoietic cells. A signaling pathway in which there is a potential role of E-cadherin is the wnt pathway and multiple lines of evidence show that this is an important growth and survival pathway in CLL [3-6]. Activation of wnt-pathway in CLL results in up regulation of target genes that are involved in cell proliferation, survival, adhesion and invasion [51]. The studies in this report confirm the association between E-cadherin and β-catenin that is able to sequester beta-catenin in the cytoplasm which has been reported earlier in other tumor model systems [12,13]. The HDACi induced E-cadherin is a thus functional protein and is able to down regulate the wnt reporter activity in three out of four CLL specimens tested. There was also a down regulation of two important downstream effectors of the wnt pathway, namely LEF and cyclin D1 genes. The growth inhibition and annexin positivity observed with HDACi in CLL specimens is thus in part also due to wnt pathway inhibition.

HDACi reverse the epigenetic modifications, reactivate gene expression and cause apoptosis in vitro in different cell types including CLL specimens [23-26]. Our own studies indicate significant apoptosis of CLL specimens with the HDACi MS-275. Studies in vivo have been reported in Tcl1 transgenic mice that develop a disease similar to CLL patients with elevated B cells, splenomegaly and infiltration of B cells in various organs [52]. The activity of HDACi AR-42 was tested in a Tcl1 transplant model and treated mice showed reduction in peripheral leukocyte counts and survived longer [53]. There are also encouraging results of clinical trial data in CLL patients with different HDACi [54]. As these inhibitors have been approved for treatment of patients with T cell leukemia [55] and are in clinical trials in other malignancies it is imperative that their mechanism of action is well understood.

## Conclusions

The role of E-cadherin gene that is silenced by epigenetic alterations and NMD mediated decay was analyzed in CLL. The silenced E- cadherin gene in CLL can be re-expressed with HDACi that increase gene transcription and also preferentially allow the expression of correctly spliced and functional E-cadherin transcripts. The mechanism by which these clinically useful drugs alter splicing patterns requires further investigations as they are being used in the treatment of a number of malignancies. The studies also indicate that besides regulating gene transcription, histone epigenetic modifications could potentially alter gene splicing patterns as well. As large number of genes are affected by epigenetic alterations in cancer they can alter splicing and the nature of the transcripts.

## Abbreviations

CLL: Chronic lymphocytic leukemia;HDACi: Histone deacetylase inhibitor;HDAC: Histone deacetylase

## Competing interest

The authors declare no competing financial interests.

## Authors’ contributions

GJ performed experiments, analyzed data and wrote the paper; WL performed experiments, analyzed data and reviewed the paper. SS performed experiments, supervised the study and wrote the paper. All authors read and approved the final manuscript.

## Pre-publication history

The pre-publication history for this paper can be accessed here:

http://www.biomedcentral.com/1471-2407/13/88/prepub
